# Supercritical Water: A Simulation Study to Unravel the Heterogeneity of Its Molecular Structures

**DOI:** 10.3390/molecules29122947

**Published:** 2024-06-20

**Authors:** Joseph Guy Gérard Ndongo Assomo, Sadollah Ebrahimi, Jean-Paul Jay-Gerin, Armand Soldera

**Affiliations:** 1 Département de Physique, Faculté des Sciences, Université de Maroua, Maroua BP 814, Cameroon; gerardnd@yahoo.fr; 2 Laboratory of Physical Chemistry of Matter (LPCM), Department of Chemistry, Faculty of Sciences, Université de Sherbrooke, 2500 Boulevard de l’Université, Sherbrooke, QC J1K 2R1, Canada; sadollah.ebrahimi@usherbrooke.ca; 3 Department of Medical Imaging and Radiation Sciences, Faculty of Medicine and Health Sciences, Université de Sherbrooke, 3001, 12th Avenue Nord, Sherbrooke, QC J1H 5N4, Canada; jean-paul.jay-gerin@usherbrooke.ca

**Keywords:** supercritical water (SCW), SPC/E molecular dynamics simulations, molecular structures, radiolysis, SCW-cooled nuclear reactors

## Abstract

(1) Background: In the quest to accurately model the radiolysis of water in its supercritical state, a detailed understanding of water’s molecular structure, particularly how water molecules are arranged in this unique state, is essential. (2) Methods: We conducted molecular dynamics simulations using the SPC/E water model to investigate the molecular structures of supercritical water (SCW) over a wide temperature range, extending up to 800 °C. (3) Results: Our results show that at a constant pressure of 25 MPa, the average intermolecular distance around a reference water molecule remains remarkably stable at ~2.9 Å. This uniformity persists across a substantial temperature range, demonstrating the unique heterogeneous nature of SCW under these extreme conditions. Notably, the simulations also reveal intricate patterns within SCW, indicating the simultaneous presence of regions with high and low density. As temperatures increase, we observe a rise in the formation of molecular clusters, which are accompanied by a reduction in their average size. (4) Conclusions: These findings highlight the necessity of incorporating the molecular complexity of SCW into traditional track-structure chemistry models to improve predictions of SCW behavior under ionizing radiation. The study establishes a foundational reference for further exploration of the properties of supercritical water, particularly for its application in advanced nuclear technologies, including the next generation of water-cooled reactors and their small modular reactor variants that utilize SCW as a coolant.

## 1. Introduction

Alongside hydroelectric power, nuclear energy holds significant potential for reducing global greenhouse gas emissions compared to coal- or gas-powered plants, making it an essential alternative to fossil fuels in the fight against climate change. The importance of this perspective was underscored during the *IAEA International Conference on Climate Change and the Role of Nuclear Power*, which took place in Vienna, Austria, during 7–11 October 2019 [1]. In recent years, significant advancements have been made in developing fourth-generation, advanced nuclear reactors, notably supercritical water-cooled reactors (SCWRs) that are central to this study. These reactors are among six innovative designs being pursued for commercial applications by the “Generation IV International Forum” (GIF) research and development collaboration [2,3,4,5,6,7,8,9,10]. The SCWR, a class of high-temperature, high-pressure, water-cooled reactors, operates above the thermodynamic critical point of water—temperatures exceeding 373.95 °C and pressures above 22.1 megapascals (MPa) for light water (H_2_O) [11]. With core inlet and outlet temperatures set at 300 °C and 600 °C, respectively, and a nominal pressure of 25 MPa, the SCWR is an extremely energy-efficient system, achieving thermodynamic cycle efficiencies of over ~45%, significantly surpassing the ~28–32% efficiencies of current conventional pressurized water reactors [2,8,9]. This high efficiency offers substantial economic benefits through lower-cost electricity generation. SCWRs are anticipated to be commercially deployed between 2030 and 2040 [2].

Over the past fifteen years, several countries have recognized the potential of SCWR technology and actively engaged in its research. This interest has driven the development of “small modular reactor” (SMR) variants of SCWRs that utilize SCW cooling [12,13], catering to the need for smaller, more flexible reactor designs that large-scale SCWRs cannot meet. These reactors aim to significantly reduce capital costs through modularization and offer potential for enhanced safety features that could minimize the consequences of severe accidents. Noteworthy among such initiatives is the *ECC-SMART project* (“European-Canada-China-Small Modular Supercritical Water Reactor Technology”), launched in September 2020 [14]. This project marks a major international collaboration, with twenty partners dedicated to advancing these energy systems and integrating them as central elements in their future energy strategies.

In this context, there is an urgent need to identify the optimal chemistry and materials under SCWR conditions [8,15]. This research is especially pivotal for the development of small modular SCWRs (SCW-SMRs), which are being considered for deployment in small remote communities and developing countries [7].

A major challenge in understanding the water chemistry of SCWRs and their SMR variants arises from limited knowledge about in-core radiolysis and the specific radiolytic species these systems produce. SCWRs operate under significantly higher temperatures and pressures—approximately 300–600 °C and 25 MPa—compared to current generation water-cooled reactors, which typically operate at temperatures of 250 to 330 °C and pressures around 10 MPa [8,15,16]. This distinct operating environment of SCWRs results in water chemistry that is markedly different from that of conventional water-cooled reactors. Bridging this knowledge gap is crucial, as it directly impacts critical factors such as material corrosion and degradation, along with the transport of corrosion products [8]. Thus, addressing this gap is essential for ensuring the long-term viability and safety of SCWRs and SCW-SMRs.

Directly measuring the chemistry within reactor cores beyond the critical point of water is exceedingly challenging, if not impossible. This difficulty primarily arises from the SCWR coolant being exposed to intense neutron and γ-radiation fields during its circulation through the reactor core. These radiation fields play a major role in contributing to the formation of various troublesome reactive oxidizing species, including hydroxyl radicals (^•^OH), hydrogen peroxide (H_2_O_2_), oxygen (O_2_, as a decomposition product of H_2_O_2_), and the superoxide anion/hydroperoxyl radical (O_2_^•−^/HO_2_^•^, depending on pH level). These species critically affect the chemical environment, operational efficiency, and aging of the reactor (see, e.g., [8,15,16,17,18,19,20,21,22,23]), potentially accelerating corrosion processes in in-core materials, particularly fuel cladding. This can lead to fuel failures and the release of fuel fragments and fission products into the coolant, which further affects the transport of radioactive materials out of the core into downstream piping components, increasing radiation exposure to reactor maintenance personnel. Furthermore, understanding the behavior of this water is crucial for predicting and mitigating corrosion processes like embrittlement, pitting corrosion, and stress corrosion cracking [8,24,25]. Addressing these issues is vital for maintaining the integrity of reactor components.

There is still limited experimental information on radiolytic yields and reaction rates in the SCW regime or at temperatures ranging from those of current subcritical water reactors to the critical temperature (see, e.g., [8] and references cited therein). Consequently, theoretical modeling and computer simulations have become essential tools for predicting the effects of water radiolysis under these extreme conditions, and their impact on materials [15,16]. In fact, the current understanding of the potential effects of water radiolysis in SCWRs or their SMR versions relies almost entirely on such models [8].

Water at high temperatures exhibits structural differences from water at ambient conditions. For instance, our recent molecular dynamics simulations demonstrated that liquid water undergoes a significant structural change around 150 °C [26]. Interestingly, this structural transformation could explain some apparent anomalies observed experimentally in the radiolysis of water at elevated temperatures. These include the thermalization distance of secondary subexcitation electrons produced in large quantities in the radiolysis of water and the rate constant for the bimolecular reaction of two hydrated electrons, both of which experience a sudden and sharp drop near 150 °C [26].

In the quest to accurately model the radiolysis of water in its supercritical state, a detailed understanding of water’s molecular structure, particularly how water molecules are arranged in this unique state, is crucial. This knowledge is especially relevant for developing Monte Carlo track-structure chemistry simulations, which rely on a precise representation of the molecular nature of the medium—all current models of radiation tracks employ a *continuum* approach to water structure, thereby neglecting the actual molecular structure [27]. An accurate description of this structure is key to advancing radiolysis studies, as it enables more reliable predictions of the yields of radiolytic species in nuclear reactor coolants [26,28,29].

Unlike ordinary water, the hydrogen bond (H-bond) network in SCWRs becomes unstable due to the high operating temperatures and pressures. This instability results in the frequent breaking and re-forming of hydrogen-bonded structures, leading to substantial density fluctuations within the water. As a result, a heterogeneous medium forms, characterized by areas of high density comprising locally hydrogen-bonded molecules arranged in diverse tetrahedral configurations, suggestive of a ‘liquid-like’ state, and simultaneously by areas of low density where molecules exist in a ‘gas-like’ state, not bonded to each other. A number of studies have helped to shed light on key parameters (see, e.g., [30,31,32,33,34,35,36,37,38,39,40,41]), yet significant gaps in knowledge persist, especially regarding the spatial arrangement of water molecules in such extreme conditions. In this context, molecular dynamics (MD) simulations have proven to be particularly valuable. They skillfully generate realistic configurations of water molecules, accurately reflecting the conditions dictated by the high temperatures and pressures characteristic of SCW. In the field of radiation science, this detailed molecular picture is essential for a deeper comprehension of how radiation energy is deposited within SCWRs [42], an aspect that remains incompletely understood.

This study builds upon prior research, where we probed the heterogeneous properties of SCW at diverse densities at 400 °C [28,29]. We now aim to employ MD simulations to conduct a detailed analysis of the structural characteristics of SCW over a temperature range of 400 to 800 °C while maintaining a constant pressure of 31 MPa at 400 °C and 25 MPa between 500 and 800 °C. These particular conditions closely correspond with the operational parameters established for SCWRs and their SCW-SMR variants [7,12,14].

## 2. Results

**Figure 1:**
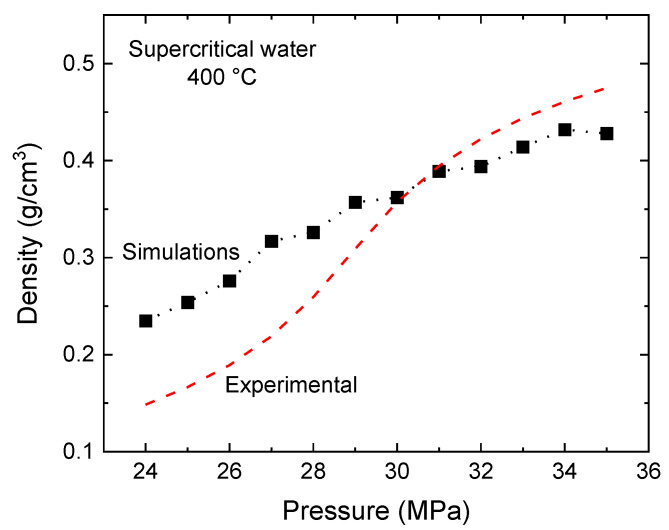
Water density (expressed in g/cm^3^) for SCW at 400 °C as a function of pressure, ranging from 24 to 35 MPa. Our simulation results are indicated by solid black squares (■), accompanied by a dotted line to aid visual interpretation. The dashed red line illustrates the experimental data, as referenced in [43].

Figure 1 illustrates the variation in water density for SCW at 400 °C across a pressure range of 24 to 35 MPa. Notably, at approximately 31 MPa, our molecular dynamics simulations align remarkably well with experimental data [43], exhibiting a minimal relative error of 1.3%. This high degree of accuracy at 31 MPa renders it an exceptionally suitable pressure for our computational analysis. In contrast, deviations from experimental densities become evident at other pressures, particularly at 25 MPa, where the error significantly increases, thereby reducing its applicability for our studies (at 400 °C). As a result, we have determined that a pressure setting of 31 MPa offers a more dependable and appropriate choice for conducting precise calculations.

To explore the molecular heterogeneity of SCW at 400 °C and the optimal pressure of 31 MPa, we analyzed the radial distribution function, *g*(*r*), with respect to the center-of-mass (COM) of water molecules. This function is defined as follows [28,29]:

(1) $$g\left(r\right)\;\;=\;\;\frac{\langle n\left(r\;\;\;\pm \;\;\frac{\delta r}{2}\right)\rangle }{4\;\pi \;r^{2}\;\rho \;\delta r\;},$$

where $n\left(r\;\pm \;\frac{\delta \;r}{2}\right)$ represents the number of molecules within a spherical shell spanning from radius *r* to $\left(r\;\;\pm \;\;\frac{\delta r}{2}\right)$, essentially within a volume of $4\;\;\pi \;r^{2}\;\delta r$. The notation $\langle \ldots \rangle$ signifies that this average is computed over a duration of 10 ns in the MD simulation. This function plays a pivotal role in quantifying the likelihood of locating water molecules at a precise distance *r* from a given reference water molecule.

**Figure 2:**
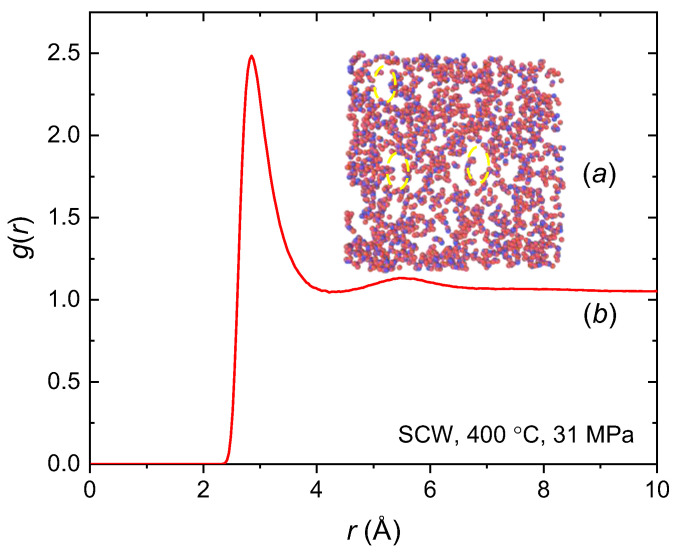
(**a**) Molecular structure of SCW at 400 °C and 31 MPa (*ρ* = 0.3945 g/cm^3^). The focus is on a 10 Å-thick slice of the simulation cell volume along the *z*-axis. The voids within this structure are outlined by a yellow dashed line. In this representation, the oxygen and hydrogen atoms of the water molecules are shown in blue and red, respectively. (**b**) Simulated COM radial distribution function *g*(*r*) of SCW at 400 °C and 31 MPa.

Figure 2 reveals a prominent peak in *g*(*r*) at a distance of 2.9 Å under the defined thermodynamic conditions of 400 °C and 31 MPa. This pronounced peak closely resembles those observed in water at temperatures below the critical point, specifically at 200, 300, and 350 °C, as documented in [26]. The origin of this peak can be traced to the first hydration shell encircling a water molecule, attributable primarily to the presence of strong hydrogen bonding within the water molecular structure. As the distance *r* increases beyond this point, *g*(*r*) shows a noticeable dip, implying the formation of void spaces. The secondary peak at ~5.5 Å indicates the presence of a neighboring molecular cluster, likely comprising 4.5 water molecules [29]. As the distance further increases, the interactions among water molecules reach a state of equilibrium, indicative of a balancing of molecular forces. This pattern, marked by alternative peaks and wells, highlights the molecular heterogeneity of SCW at 400 °C and a pressure of 31 MPa [44].

**Figure 3:**
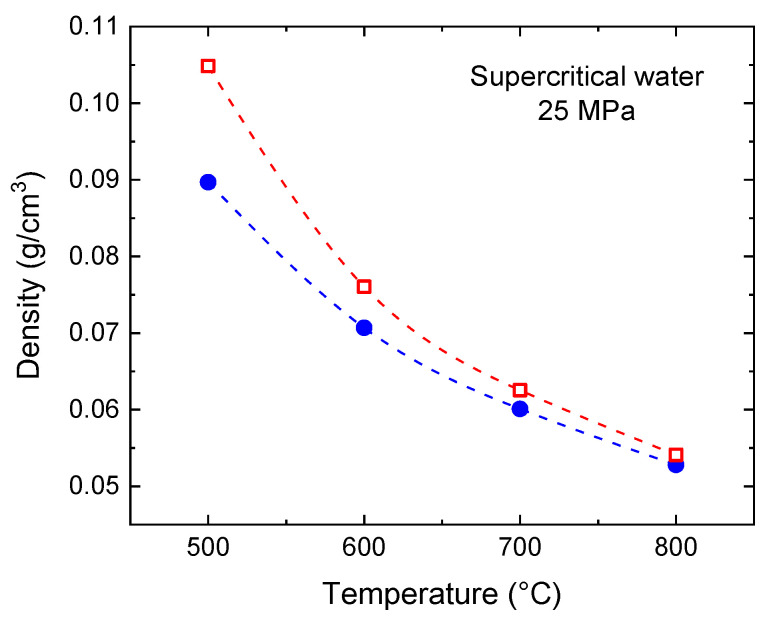
Experimental (●) and simulated (☐) densities of SCW under a constant pressure of 25 MPa. Experimental data are taken from [43]. Dashed lines serve as visual guides.

To enhance our understanding of molecular structures, we conducted a study at significantly higher temperatures in the supercritical state, specifically at 500–800 °C, and at a pressure of 25 MPa (as mentioned in the Introduction, this pressure corresponds with the operational environment chosen for SCWRs and their SCW-SMR variants). This investigation allowed us to identify and analyze the distinct molecular configurations present at these temperatures. In Figure 3, we compare the experimental and simulated densities at 25 MPa pressure. Small discrepancies are noted, which decrease as the temperature increases. Further studies to investigate local phenomena can thus be undertaken.

**Figure 4:**
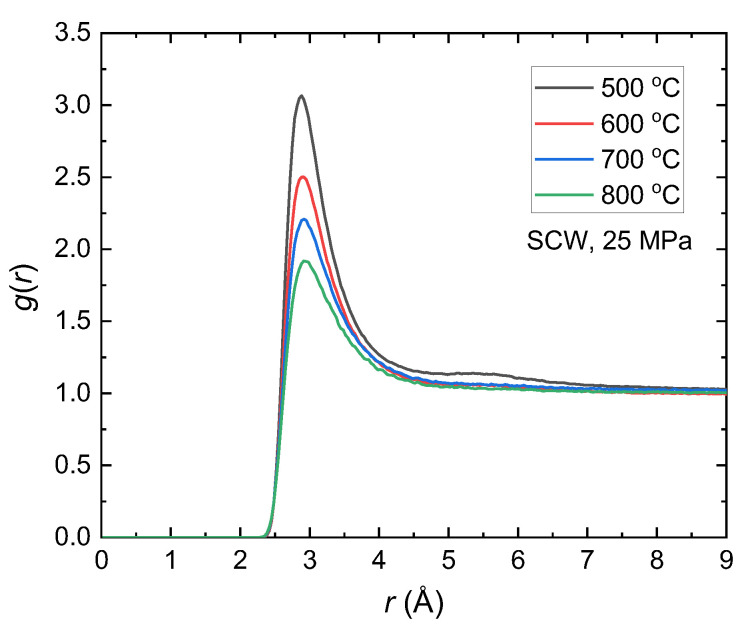
Simulated COM radial distribution functions *g*(*r*) of SCW at 25 MPa and different temperatures, each associated with its specific density. At 500 °C, the distribution is represented in black (*ρ* = 0.1048 g/cm^3^), at 600 °C in red (*ρ* = 0.076 g/cm^3^), at 700 °C in blue (*ρ* = 0.0625 g/cm^3^), and at 800 °C in green (*ρ* = 0.053 g/cm^3^).

Examination of these structures using the COM radial distribution function uncovers a uniformity in the interactions among water molecules beyond the 2.9 Å peak position, as shown in Figure 4. Moreover, in contrast to the observations made in Figure 2, under conditions of lower pressure and temperatures exceeding 400 °C, a certain instability becomes apparent in these interactions within the 4 Å to 8 Å range, denoted by the absence of a well. This lack of stability can be ascribed to the weakening of the structural integrity of the water network, which leads to the breakdown of hydrogen bonds [45,46]. As these weaker intermolecular forces break down, the molecular structure becomes less stable, leading to increased instability.

**Figure 5:**
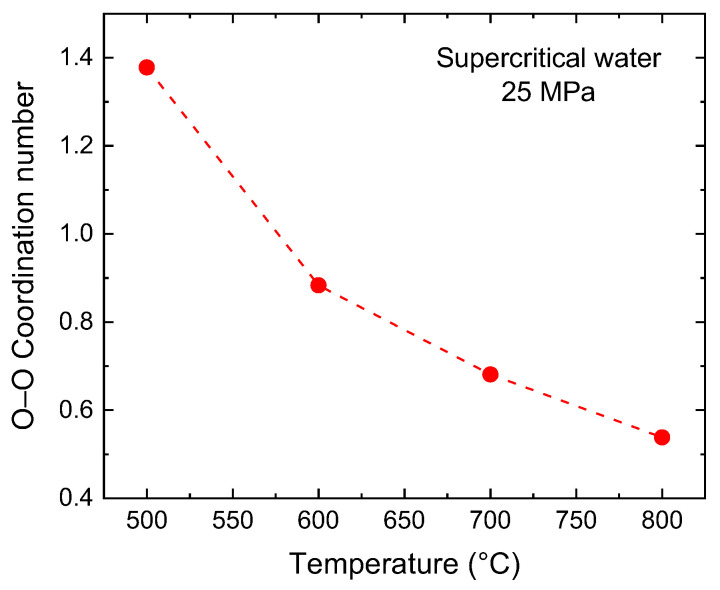
O–O coordination number as temperature increases from 500 °C to 800 °C. The dashed line serves as a visual guide.

**Figure 6:**
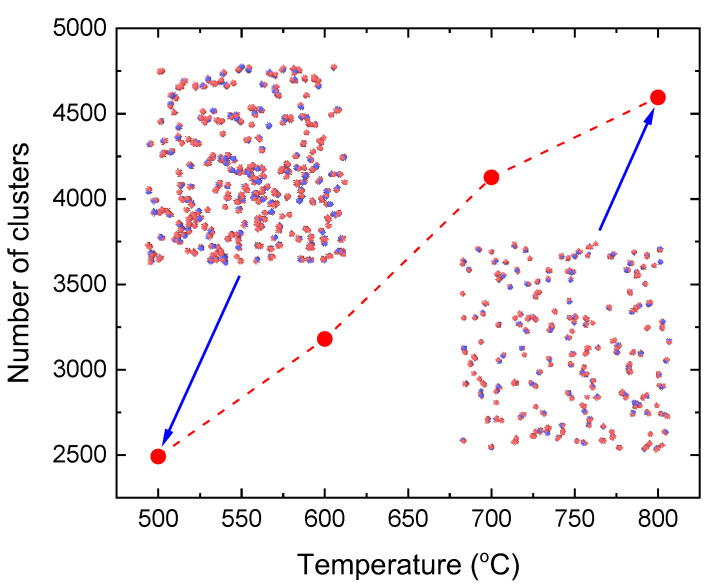
Number of clusters based on a neighbor cutoff distance of 3.5 Å in SCW at 25 MPa as a function of temperature. The dashed line serves as a guide for the eye. Snapshots at 500 °C (*ρ* = 0.1048 g/cm^3^) and 800 °C (*ρ* = 0.053 g/cm^3^) are shown. As temperature increases, the water molecules break apart (right snapshot) and eventually the size of clusters decreases. In these representations, the water’s oxygen atoms are shown in blue while hydrogen atoms are marked in red.

To unveil this behavior, an investigation into the variation of oxygen–oxygen coordination numbers in response to temperature changes provides a way to uncover the development of water molecule clusters at 500–800 °C. A key observation is the progressive decline in the O–O coordination number as temperature increases from 500 °C to 800 °C, spanning a significant range from ~1.4 at the lowest temperature to ~0.5 at the highest (Figure 5). Moreover, our findings reveal a corresponding increase in the number of clusters with increasing temperature, if we consider the cluster based on a neighbor cutoff distance of 3.5 Å, which is the maximum distance between molecules to be considered as part of the same cluster (Figure 6). This trend indicates a direct correlation between rising temperature and the reduction of coordination numbers. At lower temperatures, water molecules have less kinetic energy and cluster more closely together, forming more stable structures through hydrogen bonding. However, as temperature increases, the increased thermal motion leads to the disintegration of molecular clusters by breaking their hydrogen bonds. This disruption causes the clusters to fragment into a greater number of smaller clusters or isolated molecules, as illustrated in the snapshots shown in Figure 6. In other words, this observed decrease in cluster size, along with the reduced number of molecules linked to the coordination number at higher temperatures, could be explained by a decrease in hydrogen bonds, which is directly correlated with the O–O coordination number in the water system.

The increase in the number of clusters or the reduction in cluster size not only corroborates the formation of more atomic clusters at higher temperatures but also reflects a significant alteration in the molecular structure of water. Additionally, investigations into hydrogen bonds have revealed a decrease in their prevalence with increasing temperature, indicating significant alterations in the molecular dynamics of water under varying thermal conditions.

## 3. Materials and Methods

In our simulations, water molecules are modeled as rigid entities, with their intramolecular degrees of freedom held constant. Interaction among these molecules is treated through the extended simple point charge (SPC/E) pair potential [47]. This rigid water model is renowned for its relative simplicity and computational efficiency, making it a prevalent choice for MD simulations of water-based systems. It differs from the original simple point charge (SPC) model [48] by integrating a corrective term that reconciles the variances in polarization self-energy observed between a molecule in its liquid state and one in a vacuum.

In the SPC/E representation, a water molecule is depicted through three interaction sites: one centered on the oxygen atom and the others on the two hydrogen atomic nuclei. The O–H bond length is consistently set at 1 Å, and the H–O–H angle is maintained at the precise tetrahedral angle of 109.47°. Charge distribution is modeled with the oxygen carrying a charge of *q*_O_ = −0.8476|*e*| and each hydrogen having *q*_H_ = −*q*_O_/2 = +0.4238|*e*|, where |*e*| represents the magnitude of the electron’s charge. This arrangement ensures charge neutrality and accurately accounts for the Coulomb interactions within the system:

(2) $$U_{\mathrm{Coulomb}}\;=\;\;\frac{1}{4\;\pi \;\varepsilon_{0}}\;\sum_{i,j}\;\frac{q_{i}\;q_{j}}{r_{ij}},$$

where the summation is over all distinct pairs *i* and *j*, *ε*_0_ is the vacuum permittivity, *q_i_* and *q_j_* are the partial charges on atoms *i* and *j*, and *r_ij_* is the non-bonding distance between atoms *i* and *j* on two different molecules.

The van der Waals forces are described using a “12-6” Lennard-Jones (LJ) potential, which only concerns oxygen atoms. This potential quantifies the interaction energy between pairs of oxygen atoms, each belonging to distinct water molecules, and depends on their separation distance, denoted as *r*_OO_:

(3) $$U_{\mathrm{LJ}}\;=\;\;4\;\;\varepsilon_{\mathrm{LJ}}\;\;\left[{\left(\frac{\sigma }{r_{OO}}\right)}^{12}\;-\;\;{\left(\frac{\sigma }{r_{OO}}\right)}^{6}\right],$$

where *ε*_LJ_ = 0.6502 kJ/mol is the LJ potential well depth and *σ* = 3.166 Å is twice the van der Waals radius of oxygen.

As mentioned above, the SPC/E model further incorporates an average self-polarization energy correction term, detailed as follows [47]:

(4) $$U_{\mathrm{pol}}\;=\;\frac{{\left(\mu \;-\;\mu_{0}\right)}^{2}}{2\;\alpha },$$

where *μ* is the effective dipole moment of polarized water in the liquid state, which is 2.351 Debye (D), *μ*_0_ = 1.85 D denotes the dipole moment of the water molecule in its isolated, gas phase form, and *α* = 1.45 Å^3^ is the isotropic polarizability of water.

The total intermolecular interaction potential, *U*, for the SPC/E water model is therefore represented as the sum:

(5) $$U\;\;=\;\;U_{\mathrm{Coulomb}}\;+\;\;U_{\mathrm{LJ}}\;+\;\;U_{\mathrm{pol}}$$

The SPC/E force field, despite its simplicity, effectively replicates many of the thermodynamic and physical properties of liquid water, including diffusion coefficients as well as the dielectric constant over a wide range of temperatures and pressures [49,50]. It also reproduces, with good agreement, the liquid–vapor coexistence curve for water and predicts critical parameters, which compare favorably to the experimental values [51]. Furthermore, it has also demonstrated proficiency in accurately characterizing the structures and properties of water at high temperature and pressure conditions up to the supercritical state [26,28,29,39,40,41,42].

The simulations were conducted in accordance with the established procedure previously employed by Metatla et al. [28,29]. In summary, each simulation involved a cubic system measuring 81 Å on each side, comprising *N* = 7000 rigid water molecules, which corresponds to a total of 21,000 atoms. This system was extended in all three directions through periodic boundary conditions. The selected system size is deemed suitable for adequately representing the thermodynamic limit, thereby ensuring a proper representation of the configuration space. For handling long-range Coulomb interactions, the Ewald summation method [52] was employed and a cutoff radius of 9.5 Å was applied to the LJ interactions.

To preserve the rigid tetrahedral geometry of water molecules, the SHAKE iterative algorithm [53] was employed, applying a relative geometric tolerance of 10^−4^. Newton’s equations of motion were integrated using the velocity Verlet algorithm [52,54], and the integration time step was fixed at 1 femtosecond (fs). The simulations extended over 10^6^ steps, equivalent to a duration of 1 nanosecond (ns). All MD calculations were conducted using the LAMMPS (Large-scale Atomic/Molecular Massively Parallel Simulator) simulation package [55].

The temperature ranged from 400 to 800 °C, with the pressure held constant at 31 MPa at 400 °C and maintained at 25 MPa for temperatures between 500 and 800 °C. The initial configurations were devised using the BIOVIA Materials Studio Amorphous Cell building tool [56], set to simulate normal ambient water conditions of 25 °C and a density of 1 g/cm^3^. These configurations were then subjected to a standard energy minimization process for equilibration. This involved employing a mix of steepest descent and conjugate gradient algorithms, carried out across 5000 steps. Subsequent to this minimization, the systems were progressively heated to their respective target temperatures, increasing in increments of 25 °C, within the canonical *NVT* ensemble, where the number of molecules, the volume of the simulation cell, and the temperature remain constant. Following the heating phase, the systems were equilibrated for 100 ps at an external pressure of 25 MPa. The Nosé-Hoover thermostat algorithm [57,58] was used to maintain the systems at the desired temperature, and the Andersen barostat algorithm [59] was employed for pressure regulation. Finally, the MD simulations were performed in the isobaric–isothermal *NPT* ensemble, maintaining a constant number of molecules at constant pressure and temperature, until the system achieved an equilibrated density, a process completed over the course of 1 ns.

The molecular configurations corresponding to the range of temperatures studied, and at a constant pressure of 25 MPa, have been determined. The clusters were identified based on a selection criterion that considers the separation distance between adjacent atoms [60]. We chose a threshold of 2.9 Å for the separation distance to identify these clusters. This particular value was selected because it matches the highest molecule count in our center-of-mass (COM) radial distribution function (RDF) diagrams. This pattern is consistently observed across all the temperatures and pressures we studied.

## 4. Conclusions

The study investigated the heterogeneity of water in its supercritical state through SPC/E molecular dynamics simulations. The results indicated that at 400 °C, there is a good correlation between density values derived from theoretical and experimental methods at a pressure of 31 MPa. This finding underscores the distinct heterogeneity of water, as demonstrated by its molecular structure and the center-of-mass radial distribution function. The latter exhibits distinct molecular peaks and wells, indicating the presence of voids. A noteworthy observation is the consistency of the peak positions at 2.9 Å across all studied temperatures and pressures, mirroring patterns observed in the subcritical state. Moreover, it was observed that an increase in temperature correlates with a rise in the number of clusters formed, along with a reduction in hydrogen bonding.

This study indicates that traditional SCW radiolysis models, which consider water as a continuum, may require updates to incorporate these findings. Implementing such revisions would enhance the accuracy of predictions regarding SCW behavior under radiation. This could be important in applications like nuclear power generation and waste processing, where water is frequently utilized as a coolant and can reach supercritical states. This is especially the case for advancing our knowledge and capabilities in the context of SCWRs and their SCW-SMR variants. By better understanding the molecular interactions and transformations under these conditions, researchers and engineers can more accurately predict and mitigate the effects of radiolysis, leading to safer and more efficient reactor designs.

## Data Availability

Data generated and analyzed during this study are provided in full within the article.

## References

[1] (2019). International Conference on Climate Change and the Role of Nuclear Power.

[2] Gen IV International Forum, 2024. Supercritical-Water-Cooled Reactor (SCWR). https://www.gen-4.org/gif/jcms/c_9360/scwr.

[3] Oka Y., Koshizuka S. (1998). Conceptual design study of advanced power reactors. Prog. Nucl. Energy.

[4] (2002). A Technology Roadmap for Generation IV Nuclear Energy Systems.

[5] Schulenberg T., Leung L.K.H., Brady D., Oka Y., Yamada K., Bae Y., Willermoz G. (2009). Supercritical Water-Cooled Reactor (SCWR) Development through GIF Collaboration.

[6] Duffey R. (2016). The development and future of the supercritical water reactor. CNL Nucl. Rev..

[7] Leung L.K.H., Huang Y.-P., Dostal V., Yamaji A., Sedov A. An update on the development status of the supercritical water-cooled reactors. Proceedings of the Fourth Generation IV International Forum (GIF) Symposium.

[8] Guzonas D., Novotny R., Penttilä S., Toivonen A., Zheng W. (2018). Materials and Water Chemistry for Supercritical Water-Cooled Reactors.

[9] Pioro I. (2022). Handbook of Generation IV Nuclear Reactors.

[10] Wu P., Ren Y., Feng M., Shan J., Huang Y., Yang W. (2022). A review of existing supercritical water reactor concepts, safety analysis codes and safety characteristics. Prog. Nucl. Energy.

[11] Levelt Sengers J.M.H., Straub J., Watanabe K., Hill P.G. (1985). Assessment of critical parameter values for H_2_O and D_2_O. J. Phys. Chem. Ref. Data.

[12] (2020). Advances in Small Modular Reactor Technology Developments.

[13] Murakami T., Anbumozhi V.V. (2022). Small Modular Reactor (SMR) Deployment: Advantages and Opportunities for ASEAN.

[14] Joint European Canadian Chinese Development of Small Modular Reactor Technology (ECC-SMART). A Transcontinental Project to Bring the Potential of Supercritical Water SMRs a Step Closer to Reality, 2020. https://ecc-smart.eu/.

[15] Guzonas D., Stuart C.R., Jay-Gerin J.-P., Meesungnoen J. (2010). Testing Requirements for SCWR Radiolysis.

[16] Guzonas D., Brosseau F., Tremaine P., Meesungnoen J., Jay-Gerin J.-P. (2012). Water chemistry in a supercritical water-cooled pressure tube reactor. Nucl. Technol..

[17] Lin M., Katsumura Y., Hatano Y., Katsumura Y., Mozumder A. (2011). Radiation chemistry of high temperature and supercritical water and alcohols. Charged Particle and Photon Interactions with Matter: Recent Advances, Applications, and Interfaces.

[18] Katsumura Y., Mozumder A., Hatano Y. (2004). Application of radiation chemistry to nuclear technology. Charged Particle and Photon Interactions with Matter: Chemical, Physical, and Biological Consequences with Applications.

[19] Edwards E.J., Wilson P.P.H., Anderson M.H., Mezyk S.P., Pimblott S.M., Bartels D.M. (2007). An apparatus for the study of high temperature water radiolysis in a nuclear reactor: Calibration of dose in a mixed neutron/gamma radiation field. Rev. Sci. Instrum..

[20] Liu G., Du T., Toth L., Beninger J., Ghandi K. (2016). Prediction of rate constants of important reactions in water radiation chemistry in sub- and supercritical water: Equilibrium reactions. CNL Nucl. Rev..

[21] Liu G., Landry C., Ghandi K. (2018). Prediction of rate constants of important chemical reactions in water radiation chemistry in sub- and supercritical water– non-equilibrium reactions. Can. J. Chem..

[22] Sultana A., Meesungnoen J., Jay-Gerin J.-P. (2020). Yields of primary species in the low-linear energy transfer radiolysis of water in the temperature range of 25–700 °C. Phys. Chem. Chem. Phys..

[23] Sultana A., Meesungnoen J., Jay-Gerin J.-P. (2024). Characterizing the early acidic response in advanced small modular reactors cooled with high-temperature, high-pressure water. Radiation.

[24] Guzonas D., Cook W.G. (2012). Cycle chemistry and its effect on materials in a supercritical water-cooled reactor: A synthesis of current understanding. Corros. Sci..

[25] Macdonald D.D., Engelhardt G.R., Petrov A. (2022). A critical review of radiolysis issues in water-cooled fission and fusion reactors: Part I, Assessment of radiolysis models. Corros. Mater. Degrad..

[26] Ndongo Assomo J.G.G., Ebrahimi S., Muroya Y., Jay-Gerin J.-P., Soldera A. (2023). Molecular dynamics simulation reveals a change in the structure of liquid water near 150 °C, which may explain apparent anomalies in high-temperature water radiolysis. Chem. Afr..

[27] Green N.J.B., Pimblott S.M. (2001). Radiation track structure simulation in a molecular medium. Res. Chem. Intermediat..

[28] Metatla N., Jay-Gerin J.-P., Soldera A., Rouben B., Guzonas D., Leung L. (2011). Molecular dynamics simulation of subcritical and supercritical water at different densities. Proceedings of the 5th International Symposium on Supercritical-Water-Cooled Reactors.

[29] Metatla N., Lafond F., Jay-Gerin J.-P., Soldera A. (2016). Heterogeneous character of supercritical water at 400 °C and different densities unveiled by simulation. RSC Adv..

[30] Tucker S.C. (1999). Solvent density inhomogeneities in supercritical fluids. Chem. Rev..

[31] Kalinichev A.G., Churakov S.V. (1999). Size and topology of molecular clusters in supercritical water: A molecular dynamics simulation. Chem. Phys. Lett..

[32] Boero M., Terakura K., Ikeshoji T., Liew C.C., Parrinello M. (2001). Water at supercritical conditions: A first principles study. J. Chem. Phys..

[33] Kalinichev A.G., Cygan R.T., Kubicki J.D. (2001). Molecular simulations of liquid and supercritical water: Thermodynamic, structure, and hydrogen bonding. Molecular Modeling Theory: Applications in the Geosciences.

[34] Bernabei M., Botti A., Bruni F., Ricci M.A., Soper A.K. (2008). Percolation and three-dimensional structure of supercritical water. Phys. Rev. E.

[35] Wernet P., Testemale D., Hazemann J.-L., Argoud R., Glatzel P., Pettersson L.G.M., Nilsson A., Bergmann U. (2005). Spectroscopic characterization of microscopic hydrogen-bonding disparities in supercritical water. J. Chem. Phys..

[36] Sahle C.J., Sternemann C., Schmidt C., Lehtola S., Jahn S., Simonelli L., Huotari S., Kakala M., Pylkkänen T., Nyrow A. (2013). Microscopic structure of water at elevated pressures and temperatures. Proc. Natl. Acad. Sci. USA.

[37] Tassaing T., Garrain P.A., Bégué D., Baraille I. (2010). On the cluster composition of supercritical water combining molecular modeling and vibrational spectroscopic data. J. Chem. Phys..

[38] Swiatla-Wojcik D., Szala-Bilnik J. (2011). Transition from patchlike to clusterlike inhomogeneity arising from hydrogen bonding in water. J. Chem. Phys..

[39] Sun Q., Wang Q., Ding D. (2014). Hydrogen bonded networks in supercritical water. J. Phys. Chem. B.

[40] Skarmoutsos I., Guardia E., Samios J. (2017). Local structural fluctuations, hydrogen bonding and structural transitions in supercritical water. J. Supercrit. Fluids.

[41] Wang Y., Xu J., Ma X. (2022). Interaction between neighboring supercritical water molecules and density fluctuation by molecular dynamics simulations. J. Therm. Sci..

[42] Kallikragas D., Guzonas D., Svishchev I. (2015). Properties of aqueous systems relevant to the SCWR via molecular dynamics simulations. AECL Nucl. Rev..

[43] Lemmon E.W., Huber M.L., McLinden M.O. (2010). NIST Reference Fluid Thermodynamics and Transport Properties—REFPROP.

[44] Soldera A., Qi Y., Capehart W.T. (2009). Phase transition and morphology of polydispersed ABA’ triblock copolymers determined by continuous and discrete simulations. J. Chem. Phys..

[45] Fang Z., Xu C. (2014). Near-Critical and Supercritical Water and Their Applications for Biorefineries.

[46] Schienbein P., Marx D. (2020). Supercritical water is not hydrogen bonded. Angew. Chem. Int. Ed..

[47] Berendsen H.J.C., Grigera J.R., Straatsma T.P. (1987). The missing term in effective pair potentials. J. Phys. Chem..

[48] Berendsen H.J.C., Postma J.P.M., van Gunsteren W.F., Hermans J., Pullman B. (1981). Interaction models for water in relation to protein hydration. Intermolecular Forces.

[49] Wasserman E., Wood B., Brodholt J. (1994). Molecular dynamic study of the dielectric constant of water under high pressure and temperature conditions. Ber. Bunsenges. Phys. Chem..

[50] Kallikragas D.T., Plugatyr A.Y., Svishchev I.M. (2014). High temperature diffusion coefficients for O_2_, H_2_, and OH in water, and for pure water. J. Chem. Eng. Data.

[51] Guissani Y., Guillot B. (1993). A computer simulation study of the liquid-vapor coexistence curve of water. J. Chem. Phys..

[52] Allen M.P., Tildesley D.J. (2017). Computer Simulation of Liquids.

[53] Ryckaert J.-P., Ciccotti G., Berendsen H.J.C. (1977). Numerical integration of the Cartesian equations of motion of a system with constraints: Molecular dynamics of *n*-alkanes. J. Comput. Phys..

[54] van Gunsteren W.F., Berendsen H.J.C. (1990). Computer simulation of molecular dynamics: Methodology, applications, and perspectives in chemistry. Angew. Chem. Int. Ed. Engl..

[55] Plimpton S. (1995). Fast parallel algorithms for short-range molecular dynamics. J. Comput. Phys..

[56] BIOVIA Materials Studio 2017, Dassault Systèmes Corporate, Waltham, MA. https://www.gga.asia/upload/pdf/474/amorphous-cell_20170927140352.pdf.

[57] Nosé S. (1984). A unified formulation of the constant temperature molecular dynamics methods. J. Chem. Phys..

[58] Hoover W.G. (1985). Canonical dynamics: Equilibrium phase-space distributions. Phys. Rev. A.

[59] Andersen H.C. (1980). Molecular dynamics simulations at constant pressure and/or temperature. J. Chem. Phys..

[60] Stukowski A. (2010). Visualization and analysis of atomistic simulation data with OVITO—The Open Visualization Tool. Modelling Simul. Mater. Sci. Eng..

